# Improving aerobic capacity in patients with advanced non-small cell lung cancer: study protocol of the 3-armed randomized controlled BREATH trial

**DOI:** 10.1186/s13063-025-09416-2

**Published:** 2026-01-17

**Authors:** Nico De Lazzari, Marcel Wiesweg, Miriam Götte, Jan Franco, Raluca Ileana Mincu, Johannes Jäger, Eva-Maria Huessler, Nils Kuklik, Andreas Stang, Matthias Totzeck, Mitra Tewes

**Affiliations:** 1 Department of Palliative Medicine, West German Cancer Center, University Hospital Essen, Essen, 45147 Germany; 2 West German Cancer Center, University Hospital Essen, University of Duisburg-Essen, Essen, 45147 Germany; 3 Department of Medical Oncology, West German Cancer Center, University Hospital Essen, Essen, 45147 Germany; 4 Institute for Medical Informatics, Biometry and Epidemiology, Medical Faculty, University of Duisburg-Essen, Essen, Germany; 5 Centre for Clinical Trials Essen, University Hospital Essen, Essen, Germany; 6 Department of Cardiology and Vascular Medicine, West German Heart and Vascular Center Essen, University Hospital Essen, University of Duisburg-Essen, Essen, Germany; 7 Pediatric Clinic II, Department of Pediatric Nephrology, Gastroenterology, Endocrinology and Sonography, University Hospital Essen, University of Duisburg-Essen, Essen, Germany

**Keywords:** Advanced lung cancer, NSCL, Exercise, Palliative treatment, VO_2_ peak

## Abstract

**Background:**

Lung cancer is one of the most common cancers in Germany, with around 56,000 new cases diagnosed in 2020. Approximately 65% are diagnosed at advanced stages, where symptoms such as fatigue, pain, dyspnea, and weight loss are prevalent. These patients often suffer from cardiovascular and pulmonary comorbidities, which interact with treatment toxicity, outcome, and increase treatment costs. Although exercise therapy is proven to alleviate cancer-related symptoms and to improve quality of life, current lung cancer treatment guidelines fail to adequately prioritize its crucial role.

**Methods:**

The Better symptom contRol with Exercise in pAtients wiTH advanced non-small cell lung cancer (BREATH) study is a prospective, three-arm randomized controlled trial (RCT) designed to assess the impact of exercise therapy on patients with advanced NSCLC (stage IIIB-IV) who are receiving first- or second-line systemic therapy in the palliative setting. Patients (*n* = 104) are randomized in a 2:1:1 ratio into a control group (receiving exercise recommendations) or one of two intervention arms: endurance training and breathing exercise or combined endurance and resistance training. The intervention groups will exercise twice a week for 12 weeks. The control group participants will be randomized again in a 1:1 ratio into one of the two intervention arms after completion of the control period. The study will assess outcomes at baseline, 12 weeks, and 24 weeks. The primary outcome is improvement of aerobic capacity (VO_2_ peak). Secondary outcomes include quality of life, fatigue, adherence to exercise, and adverse events. Patient representatives were involved in all stages of protocol development.

**Discussion:**

The BREATH study addresses a significant gap in the current management of advanced lung cancer treatment by evaluating the impact of different exercise treatment protocols to reduce symptoms and improve clinical outcome. The study design and exercise program aim to enhance adherence and optimize patient-related outcomes. The results of the BREATH study have the potential to influence future guidelines and improve the management of patients with advanced NSCLC.

**Trial registration:**

ClinicalTrials.gov NCT06374160. Registered on April 18, 2024.

## Background

With approximately 56,000 new cases in 2020, lung cancer is one of the most common cancer entities in Germany [1]. Approximately 65% are diagnosed in advanced stages [2–4]. Prominent symptoms during the cancer trajectory include tumor-related fatigue [5, 6], pain [7], depression [8], cough [9], dyspnea (shortness of breath) [10], weight loss/appetite loss [11], and sleep problems [12, 13]. In addition to these cancer-related symptoms, patients with advanced lung cancer typically present with comorbidities, with the common causal root in nicotine consumption. Cardiovascular diseases and chronic obstructive pulmonary disease (COPD) form the most common and relevant comorbidities [14]. Besides a significantly adverse effect on treatment outcome [15], patients with multiple comorbid conditions require more outpatient and inpatient treatments, leading to higher treatment costs [16, 17]. To support these multimorbid patients, multimodal strategies need to be developed. The Roundtable Report of the American College of Sports Medicine recommends that cancer patients engage in moderate physical activity for 150–300 min per week [18]. However, in the current German S3 guidelines for the diagnosis and treatment of lung cancer, exercise interventions are only marginally addressed [2], and in the real-world setting, oncologists rarely recommend exercise [19]. Additionally, many barriers exist for patients to self-implement exercise as a routine. Over 700 studies involving 50,000 cancer patients have evaluated the impact of exercise therapy in oncological patients [20]. Exercise therapy demonstrates improvements in cancer-related side effects such as fatigue, pain, quality of life [21], dyspnea [22–24], cardiologic performance [25] or physical fitness, and prevents muscle loss during active systematic treatment [26–31]. In addition to improving cancer-specific symptoms, exercise therapy interventions show a positive impact on patients with COPD or cardiovascular disease [32–34]. Since comorbidities have been proven to worsen the quality of life and prognosis of therapy, exercise therapy can offer relief from symptom burden in addition to oncological systematic therapy [35–37]. Advanced metastatic lung cancer patients exhibit a significantly increased symptom burden along with a low quality of life compared to other tumor entities [38]. The feasibility of exercise and potential barriers in patients with advanced cancer have been studied in recent years [39–41]. Published exercise studies specifically targeting patients with advanced lung cancer have limited evidence: sample sizes in clinical trials were insufficient to evaluate the effect of exercise therapy. Almost all randomized controlled trials included fewer than 50 subjects [42–55]. Recruitment rates range from 25 to 70%, with significantly higher dropout rates (15–42%) compared to other patient cohorts with advanced tumor diseases. Only half of the published studies report the rate of adherence to the exercise intervention.

First-line treatment of metastatic non-small lung cancer (NSCLC) is molecularly stratified. After molecular diagnostics, eligible patients (EGFR, ALK, ROS1, RET) receive targeted treatment, while the majority of patients receive (chemo)immunotherapy. Standard-of-care platinum-based chemo-immunotherapy typically consists of four cycles of platinum-containing chemotherapy combined with immunotherapy, followed by immunotherapy maintenance. The addition of immunotherapy in the last decade led to a significant improvement, with 5-year overall survival rates of 20%, or 30% in selected subgroups [56, 57]. Despite this improvement in therapy, there are currently no studies combining standard-of-care immunotherapy-based systemic therapy for advanced lung tumors with exercise intervention. Of note, immune checkpoint inhibitors (ICI) targeting programmed cell death protein 1 (PD-1), programmed cell death protein ligand 1 (PD-L1), or cytotoxic T-lymphocyte-associated protein 4 (CTLA-4) have the potential to induce rare but severe toxicity such as pneumonitis, hepatitis or ICI-related myocarditis, heart failure and arrhythmias, other various cardiologic diseases, or major cardiac events (MACE) [37, 58–63]. Initial preclinical studies show a positive synergistic effect between exercise therapy and immunotherapy [64–68]. Considering the current literature, it is evident that a successful exercise therapy concept has not yet been implemented for advanced lung cancer patients.

### Objectives

The primary objective is to achieve an improvement in aerobic capacity (VO_2_ peak) from t0 to t1 through exercise therapy compared to the standard of care. As a secondary objective, we aim to examine whether there is a difference between the two exercise therapies regarding the performance improvement (VO_2_ peak) from the beginning of the therapy to the end of the therapy (either t2 compared to t1 or t1 compared to t0, depending on the randomization arm). Other secondary objectives are to assess changes in quality of life, fatigue, dyspnea during the study participation and evaluation adherence to the intervention, dropout rates, adverse events, and tumor therapy response.

## Methods and design

**Fig. 1:**
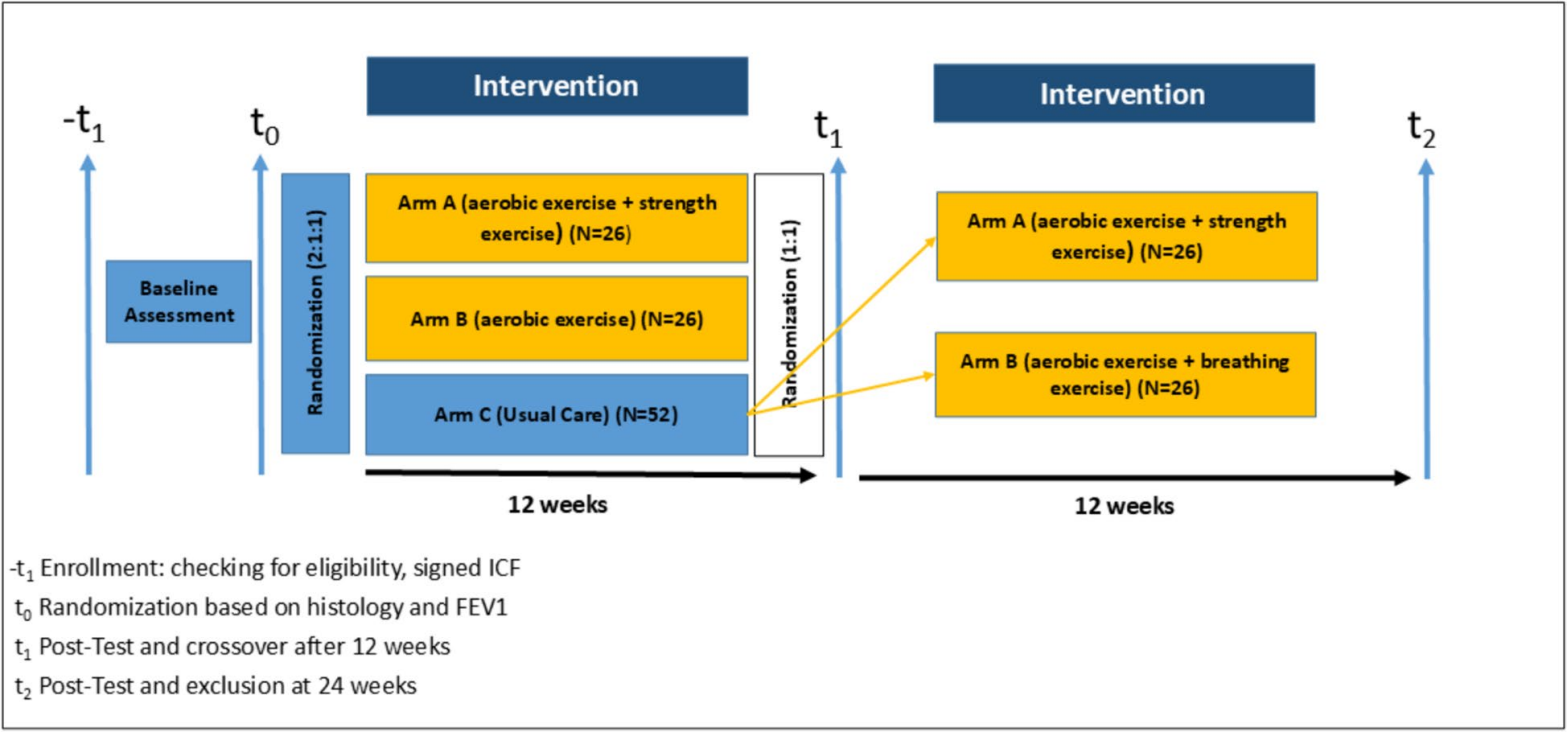
Study design of the BREATH study

BREATH is a prospective 3-arm randomized controlled trial (RCT) including histologically confirmed advanced NSCLC (UICC stages IIIB, IIIC, and IV) patients during palliative anti-cancer treatment. Patients in stage IIIB and IIIC will only be enrolled if potentially curative treatment options were excluded. Enrollment started in June 2024 and is estimated to be open for 2.5 years (Fig. 1). Study participants will be divided into study arms using a 2:1:1 randomization, with one control group and two exercise therapy groups. Participants in the two intervention arms will undergo exercise therapy, with one group receiving individual endurance training with additional breathing exercise and the other group receiving a combination of endurance and resistance training. Both intervention groups will exercise supervised twice a week for 12 weeks. The control group will initially undergo standard of care for 12 weeks. Afterwards, the patients in the control group will be randomized into one of the two intervention arms and will also receive treatment twice a week for 12 weeks. This approach has the advantage of providing a sufficiently large sample for both comparing overall exercise therapy with the control group and comparing the two types of exercise therapy. In addition, this design allows all participating patients to receive exercise therapy. By individually measuring the hypothetical one-repetition maximum (h1-RM) and conducting cardiopulmonary exercise testing (CPET) on a cycle ergometer, a tailored individual training program based on current performance will be offered to the patient. BREATH exercise begins with moderate exertion and is progressively increased during the course of the study participation (see Interventions for a detailed description). Outcome measurement will be at baseline (t_0_), after 12 weeks (t_1_), and after 24 weeks (t_2_), leading to a maximum study participation of 6 months (Fig. 1). The Ethics Committee of the Medical Faculty University of Duisburg-Essen approved this study (22–10522-BO) in December 2023 (Table 1).

**Table 1:** Inclusion criteria of the BREATH study

Inclusion criteria	Exclusion criteria
• Patients with histologically confirmed non-small cell lung carcinoma in UICC stages IIIB, IIIC, and IV • First- or second-line therapy (inclusion up to 28 days after the first cycle) in palliative intention • Age ≥ 18 years • Signed informed consent	• Severe cardiopulmonary disease (EF < 30%) • Newly occurring or progressive uncontrolled CNS (central nervous system) metastases • Expected life expectancy < 3 months • Bone metastases with acute risk of fracture • ECOG (Eastern Cooperative Oncology Group) performance status > 2 • Acute pulmonary embolism • Acute myocardial infarction • Requiring surgery for aortic aneurysm • Tension pneumothorax • Lack of proficiency in the German language • Active infection

Patients with NSCLC at the West German Cancer Center (University Hospital Essen and University Medicine Essen—Ruhrlandklinik) will be identified by the treating physicians, specialist nurses, by screening patients presented at the multidisciplinary tumor board, or by systematically screening patients receiving routine follow-up scans during first-line treatment. Furthermore, patients will be recruited through clinic posters, study flyers, and direct outreach during visits to local lung cancer self-help groups. Informed consent will be obtained by the treating physician. After study inclusion, exercise assessment (baseline) followed by randomization will be performed. Intervention will be delivered by exercise scientists with a special qualification in oncological exercise therapy.

Slow recruitment will be addressed by intensifying on-site outreach (additional posters/flyers, repeated visits to the self-help group, and regular reminders to treating physicians).

### Participant retention and follow-up

Unless patients withdraw consent entirely, we will continue to invite them to all scheduled assessments and collect the primary outcome (VO_2_ peak at 12 weeks and 24 weeks) and secondary outcomes (patient-reported outcomes). Data collected up to the point of discontinuation or death will be retained for ITT and PP analyses.

### Variables and data collection

The full variables of the BREATH study are displayed in Table 2. Anthropometric and medical data includes the following variables: age, weight, highest education, marital status, date of first cancer diagnosis, tumor diagnosis, tumor stage, TNM classification, first-line or second-line treatment, histology, metastasis and location, PD-L1 expression, and molecular pathology based on sequence analyses.

**Table 2:**
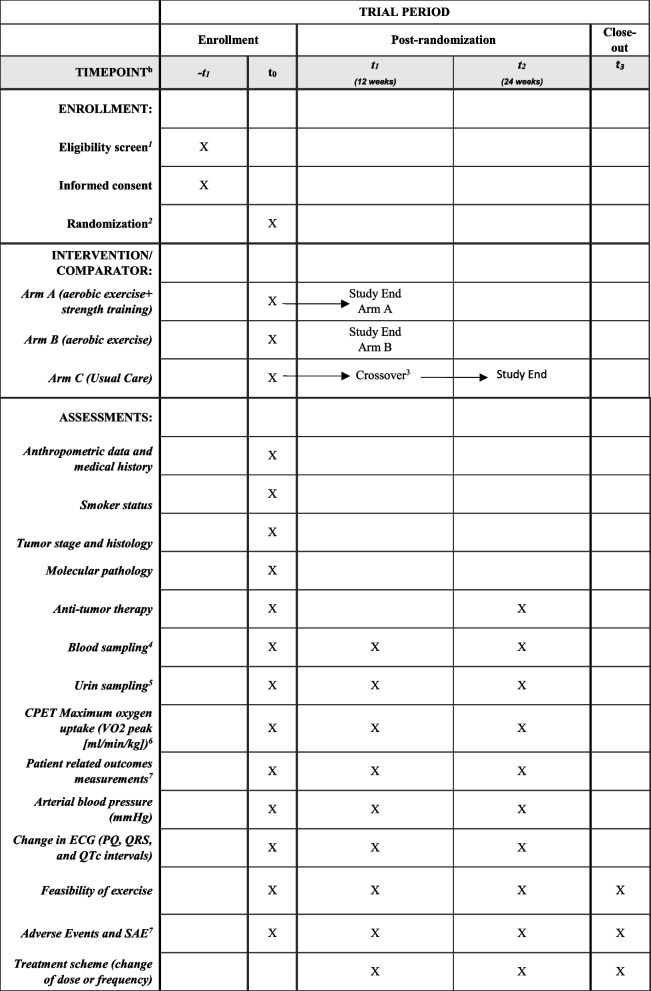
Spirit figure of the trial data collection points

^1^Check Table 1 for inclusion criteria

^2^Allocation to intervention groups will be done after completion of baseline assessment. Following randomization, strata will be used: 1. Histological stratification (non-squamous vs. squamous) and 2. Forced expiratory volume in 1 s (FEV1) >50 and under <50

^3^Randomization of the control group based on the same strata used for the first randomization

^4^Blood sampling will be done at every visit for anti-cancer treatment mostly every 3 weeks until the study ends. Cisplatin-based treatments 1 + 8 day followed by 3 weeks. Carboplatin and docetaxel d1 q3w. Blood sampling consists of BNP, high-sensitive troponin T/I as heart markers, hemoglobin [g/dl], erythrocytes [count/pl], leukocytes [count/nl], lymphocytes [count/nl], neutrophils [count/nl], C-reactive protein [mg/dl], and cytokeratin-19 fragment, CYFRA 21-1 [ng/ml]

^5^Urine (3 mL) and blood (1.5 mL) (EDTA anti-coagulated) will be collected for nuclear magnetic resonance (NMR)-based metabolome analysis

^6^Full variable of CPET consisting of forced vital capacity [L], maximal voluntary ventilation [L/min], forced expiratory volume in 1 s, one-second capacity [L], relative one-second capacity, Tiffeneau index [%], pmax [watt], HR [beats/minute], BP [mmHg], O2 saturation [%], breathing reserve [%], ventilatory threshold 1 [ml/min], ventilatory threshold 2 [ml/min], max O2 pulse [ml/beat], ventilatory equivalent for carbon dioxide slope, partial pressure in the arterial blood [mmHg], O2 partial pressure in the arterial blood [mmHg], dead space volume/tidal volume, P(A-a)O2, oxygen consumption/work load, respiratory exchange ratio (RER), pH value, PAO2 [mmHg], SaO2 [%], PCO2 [mmHg], BE [mmol/l], HCO3 [mmol/l], and SBCe [mmol/l]

^7^To be filled out by patient: EORTC-QLQ-C30—lung cancer specific LC13 and Functional Assessment of Cancer Therapy-Fatigue Scale

^8^Adverse events/serious adverse will be collected throughout the study. AEs are documented weekly by the treating physician, exercise therapist, or the study nurse using the manual of Common Terminology Criteria for Adverse Events (CTCAE) v5.0 and CTCAE after the cardio-oncology guidelines. All adverse events will be documented weekly, whereas any serious adverse event will be reported immediately (within 24 h) to the principal investigator, forwarded to the Ethics Committee and the Trial Steering Committee according to institutional requirements, collected irrespective of relatedness to exercise, and followed by appropriate clinical management by the treating team

Tobacco use plays a major role in the development of lung tumors and poorer cardiopulmonary performance. To fully understand this variable, the following items are asked: pack years and current smoking status (current smoker, non-smoker, and former smoker). A former smoker is defined as a person who has quit smoking for longer than the last 12 months. To capture this category, the abstinence date is also queried. The dose of cigarettes smoked is categorized as light smoker (persons who smoke a low number of cigarettes per day, typically less than 10 cigarettes), moderate smoker (persons who smoke a moderate number of cigarettes per day, typically between 10 and 20 cigarettes), and heavy smoker (persons who smoke a high number of cigarettes per day, typically more than 20 cigarettes).

In general, a comparison for all primary and secondary outcomes will be conducted between both exercise arms and the control arm and additionally between the two exercise arms (either t_2_ compared to t_1_ or t_1_ compared to t_0_, depending on the randomization arm).

### Randomization

Patients will be randomized into one of three study arms (arm A: endurance + resistance training, arm B: endurance training + breathing exercises, arm C: control group = usual care + exercise recommendation (following the guidelines of the American College of Sports Medicine)) using a web-based tool (https://randomizer.at). The allocation ratio is 2:1:1 for the first randomization into the control arm and two exercise arms followed by a 1:1 randomization of the original control group. Randomization is conducted after baseline assessment and is stratified based on the following two criteria to ensure balanced distribution of prognostic factors across all groups:

Histological stratification (non-squamous vs. squamous)

Squamous and non-squamous lung cancer differs by underlying biology, molecular characteristics, and response patterns to systemic therapy.

Forced expiratory volume in 1 s (FEV1)

FEV1 is a crucial measurement of lung function and is highly associated with VO_2_ peak. In advanced lung cancer patients, impairment of pulmonary function is common. FEV1 will be divided into two classifications: mild to moderate COPD—FEV1 is equal to or greater than 50% of predicted value and severe to very severe COPD—FEV1 is lower than 50% of predicted value.

The minimization method is used for stratified sampling [69]. Due to the nature of the intervention, participant blinding is not feasible. However, allocation concealment is ensured because the randomization sequence is generated and implemented through a secure, web-based system that prevents foreknowledge of group assignment. Outcome assessors conducting VO_2_ peak testing will remain blinded to treatment allocation. The randomization sequence was generated by an independent statistician. After baseline assessment, the responsible exercise scientist randomizes participants to the intervention groups using the secure, web-based randomization tool.

### Cardiopulmonary exercise testing

CPET will be carried out to assess the patient’s cardiopulmonary performance using the cycle ergometer in a semi-recumbent position. Through a breathing mask, inhaled and exhaled air can be analyzed, enabling a precise calculation of oxygen uptake (VO_2_) and carbon dioxide output (VCO_2_). To assess participants’ peak oxygen uptake (VO_2_ peak), a ramp protocol will be utilized. This diagnostic procedure is characterized by high precision in determining the anaerobic threshold through the ratio of VO_2_ to VCO_2_. The VO_2_ peak is considered a crucial factor in aerobic performance. Cardiopulmonary exercise testing also proves to be a safe diagnostic tool for advanced lung cancer patients [70].

### Physical function based on the hypothetical 1-repition maximum (h1-RM)

The one-repetition maximum (1RM) is a test to determine the maximum weight that the patient can move within a predefined range of motion. The test procedure for measurement of maximum strength is characterized by high reliability. As the one-repetition maximum represents an increased risk of injury in patients, there is the possibility of calculating the hypothetical one-repetition maximum (h1-RM). For this purpose, the Brzycki formula is used, and the maximum force can be calculated from this after the exercise and reduce the risk of injury or other adverse events [71].

### Questionnaires

The Functional Assessment of Chronic Illness Therapy—Fatigue (FACIT-Fatigue) questionnaire comprises 13 questions aimed at assessing self-reported fatigue and its impact on daily activities and physical function [72, 73]. The EORTC QLQ-C30/LC13 is a tumor entity-specific questionnaire designed to assess the quality of life in patients with lung cancer. The EORTC QLQ-C30/LC13 captures functional parameters (physical functioning, role functioning, cognitive functioning, emotional functioning), symptom scales (pain, fatigue, nausea, and vomiting), Global Quality of Life, and Global Health Status. Additionally, patients can indicate individual items that were symptomatic in the past week [74, 75]. The questionnaires are self-administered by patients at each time point; if study personnel need to assist, it will be documented in the trial’s management.

### Exercise adherence

Feasibility parameters including recruitment rate, dropout rate, and adherence to the training will be measured and documented by the responsible exercise therapist. All withdrawals will be considered as dropouts, with reasons noted and compared between each study arm. This provides a straightforward comparison of the raw dropout counts between groups. The percentage of participants who dropped out will also be calculated for each study arm relative to the total number of participants initially assigned to that arm. Adherence based on the absolute numbers of scheduled exercise sessions, both absolute and percentage-based adherence can be calculated and compared between study arms. Adherence to the protocol is defined as completing at least 75% of the possible training sessions.

### Interventions

Intervention (arm A) is a supervised exercise program combining endurance training and resistance training. Participants conduct two exercise sessions each week, with each session lasting 60 min. The breakdown of these sessions involves 20 min dedicated to endurance training and 40 min to resistance training. Aerobic interval training is characterized by an intensity set at 50% of the participants’ maximal workload, determined through CPET. This approach aims to optimize cardiovascular fitness through controlled and targeted aerobic exercises. Resistance training targets major muscle groups during each session. The exercise protocol prescribes two sets of each resistance exercise, with participants completing 8–12 repetitions per set. The training intensity for resistance exercises is progressively increased within a range of 50–80% of the participants’ h1-RM. This tailored approach allows for a progressive and adaptive response, ensuring that participants are appropriately challenged while minimizing the risk of overexertion. The initiation phase, scheduled for week one, is characterized by an intentional starting point at 30% of participants’ maximum capacity. The primary objective during this initial phase is to prepare the muscles for subsequent exertion while concurrently fostering an increased awareness of the body’s responses to the introduced exercise stimuli.

Arm B focuses exclusively on supervised aerobic exercise followed by breathing exercise. Participants in arm B engage in exercise twice per week, with each session lasting 30 min of intensity interval training. The endurance training in arm B adopts an interval-based method to strike a balance between exertion and recovery. The training intensity is set at 50% of the participants’ maximal workload and is increased during the intervention to 80% of maximal workload. The structured intervals involve 10 sets of 2 min of exertion alternated with 10 sets of 1 min for recovery, resulting in a total exercise time of 30 min per session. This design aims to challenge participants’ aerobic capacity while providing adequate recovery intervals. Following the aerobic intervals, participants perform 5 min of supervised pursed-lip breathing under the guidance of an exercise scientist, completing approximately 8–12 breathing cycles per minute three times. Additionally, patients will be performing 3 sets of 30 s of planks.

A pulse oximeter is employed for measuring heart rate and oxygen saturation to ensure safety. Additionally, the Borg-CR scale is utilized to assess subjective dyspnea after every second interval, providing a qualitative measure of perceived exertion and discomfort. Termination criteria are established to guide the conditions for stopping exercise within treatment arms: oxygen saturation <85% without increase during recovery and subjective increase in Borg-CR scale indicating severe dyspnea.

The control group receives a one-time physical activity consultation with general information about daily activities and exercise participation, as well as individual training recommendations. After 12 weeks, the control group will be randomized into one of the exercise groups.

#### Concomitant care and co-interventions

All participants will continue to receive standard medical care throughout the study, and no clinically indicated treatment will be restricted. All medications will be documented using ATC codes to enable transparent post hoc evaluation of potential influences on exercise capacity or cardiopulmonary outcomes. Nutritional intake—including oral nutritional supplements, protein products, or energy-dense formulations—will not be restricted, acknowledging the high prevalence of cancer-associated weight loss and the ethical necessity of supporting adequate nutrition in this population. Existing physiotherapy or rehabilitation programs may continue if they were established prior to enrollment and remain unchanged in frequency, type, and intensity. To minimize contamination of the intervention effect, participation in other exercise trials and the initiation of high-intensity exercise programs outside the study period are discouraged but not prohibited. This approach ensures both ethical feasibility and scientific transparency. Overall, the trial prioritizes comprehensive documentation rather than restriction of concomitant care to reflect real-world clinical practice in advanced lung cancer.

### Metabolic fingerprinting

Venous blood (EDTA) and urine samples from all participants are collected at the onset of the first session and subsequently every 12 weeks until the conclusion of the study. Both sample types are subjected to centrifugation at 900 ×g at 4 °C for 10 min, and the resulting supernatant is stored at −80 °C for future analysis.

For metabolomic analysis, the samples are diluted with specific urine and plasma diluents, and sodium trimethylsilylpropanesulfonate is added as an NMR standard. NMR-based metabolomics provides a quantitative platform to profile baseline metabolic alterations in advanced lung cancer and to assess how exercise modulates systemic pathways (e.g., amino acid, lipid, and energy metabolism) in order to explore links between metabolic patterns, physiological adaptation, and clinical outcomes. Laboratory analyses are restricted to non-genetic biochemical and physiological measurements. No genomic, germline, or whole-genome analyses will be performed. Laboratory staff receive only a pseudonymized participant ID, with no access to identifiable data. All procedures follow institutional governance and quality-control standards. There is no planned future use of samples within the main study. Samples will be analyzed only for the metabolic evaluations described in the protocol, and no additional storage for other research is intended. Some patients, however, have signed a separate optional consent allowing their samples to be used in a related translational research project (e.g., potential proteomic analyses/metabolomics). Any such work will be conducted under a separate ethics approval and according to applicable governance requirements.

### Evaluation of the intervention and patient-centered interviews

Quantitative and qualitative data are collected as part of the evaluation process [76]. The quantitative component focuses on participants’ satisfaction with various aspects of the exercise intervention. A self-developed online questionnaire assesses elements such as the structure and delivery of the intervention, organizational aspects, and support provided by the trainer team. Responses are recorded on a five-point Likert scale (“strongly disagree” to “strongly agree”), and sociodemographic information is also collected. Open-ended items allow participants to elaborate on their responses and provide detailed feedback. The survey is distributed to all participants, and data are analyzed descriptively. Frequency distributions, means, medians, standard deviations, and modes describe satisfaction levels and variability. Subgroup comparisons (e.g., age, intervention format) help identify potential differences. Free-text responses are analyzed using qualitative content analysis [77, 78], enabling systematic coding and inductive category development.

The qualitative component consists of episodic interviews with a purposive sample of approximately 15 participants. While participants are comparable in terms of their underlying medical condition, the sample is deliberately selected to reflect diversity in other relevant characteristics. Selection is based on factors such as age, gender, and symptom burden to capture a broad range of experiences.

The number of interviews is guided by data saturation and may be adjusted as needed. The interviews explore perceived changes in quality of life, physical well-being, self-efficacy, and sustained physical activity. The episodic format captures both specific experiences and broader reflections. Transcripts are analyzed using qualitative content analysis according to Kuckartz [77], a method well suited to identifying thematic patterns in narrative data.

Findings from both components are expected to inform the development and refinement of future exercise interventions. Combining structured satisfaction data with personal narratives supports improvements that are both evidence-informed and participant-centered [79, 80].

### Sample size calculation

The sample size calculation is based on the primary endpoint of the improvement in VO_2_ peak after 12 weeks (t_1_) compared to baseline (t_0_). Using a study by Quist et al. [81], where VO_2_ peak was measured in patients with advanced lung cancer before and after a 6-week exercise therapy, an assumed mean difference of 0.1 is considered between the exercise therapy groups and the control group. Additionally, a standard deviation of 0.18 is assumed [80]. With a one-sided *t*-test assuming equal variances at a significance level of 0.05 and a power of 0.80, 41 patients are required in the control group and 41 patients in the exercise therapy groups. Considering that approximately 15% of deaths are expected after 24 weeks due to disease progression [82], a dropout rate of 20% is assumed, resulting in the inclusion of 52 patients per group (52 patients in the control group, 26 patients in arm A, and 26 patients in arm B). The 52 patients of the control group will also be randomized to one of the treatment arms, resulting in also 52 patients per treatment arm for the comparison of arm A vs. arm B for the secondary objective. All secondary analyses, including the secondary objective, are exploratory, so that the sample size calculation is only based on the primary objective.

### Data analysis

Statistical analyses of the primary and secondary endpoints will be conducted on the intention-to-treat (ITT) population. As a sensitivity analysis, the analyses will also be performed on the per-protocol (PP) population. The primary endpoint of the improvement in VO_2_ peak after 12 weeks (t_1_) compared to baseline (t_0_) will be assessed using a one-sided two-sample *t*-test assuming equal variances at a significance level of *α* = 0.05. In addition, a linear regression will be applied, adjusting for age, sex, non-squamous vs. squamous, and FEV1. All secondary analyses, including the secondary objective, will be exploratory, meaning without adjustment of the significance level for multiplicity, and will be conducted using standard methods of statistical inference. Quantitative variables (including test scores) will be summarized by mean and standard deviation and analyzed using univariable and multivariable linear regression to detect differences between the groups. Dichotomous variables will be summarized by percentage frequencies and analyzed using a univariable and a multivariable generalized linear model with identity link in order to estimate absolute risk differences. Event times will be summarized by percentage frequencies analyzed using univariable and multivariable Cox regression, and Kaplan-Meier curves will be illustrated for event times. Subgroup analyses will be conducted based on age, gender, and first- or second-line treatment. Compensation for any study-related harm is provided through trial-specific patient insurance. Missing outcome data because of death or worsening condition will be treated as the worst case scenario. If, in addition, no more than 5% of the primary objective (or secondary objective) endpoint are missing, no additional imputation will be performed. If more than 5% of the primary objective (or secondary objective) endpoint are missing for other reasons than death or worsening, the analysis will be based on multiple imputation assuming MAR (missing at random). Details of the statistical analyses including sensitivity analyses will be specified in a Statistical Analysis Plan (SAP) prior to the start of analysis. Due to the study design with a wait-and-watch control group that later transitions into the exercise intervention, full blinding of data analysts is not feasible. However, treatment groups will be coded using neutral labels (e.g., group 1 and group 2), and analyses will be performed without access to identifiable clinical data.

The processing of NMR-based metabolomic data will be performed using an adapted version of the AlpsNMR package in R, which includes baseline correction, spectra alignment based on sodium trimethylsilylpropanesulfonate (DSS), and total intensity normalization [83]. The statistical analyses will include principal component analysis (PCA) with robust outlier detection. Loadings between |0.7| and |1| will be employed to identify the main determinants of the observed changes in the blood metabolome. The identification of the underlying substances in the PCA will be conducted using the Human Metabolome Database (https://hmdb.ca/). Furthermore, partial least squares (PLS) analysis will be used to correlate these changes in the metabolome with other clinical and biochemical parameters, thereby identifying the main drivers of plasma metabolome changes. Pearson correlation will be employed for numeric data with a linear relationship and normal distribution, while Spearman correlation will be used for rank data or non-linear relationships. Correlations between 0.7 and 1.0 (or −0.7 and −1.0) are generally considered strong, while correlations between 0.4 and 0.7 (or −0.4 and −0.7) are moderate. Correlations with a *p* value less than 0.05 are considered statistically significant.

Metabolic data analysis utilizing the R package ALPs was modified by researchers at the Children’s Hospital 2, University Hospital Essen, to meet the particular requirements of this study.

### Data security and storage

During the study period, the pseudonymized clinical data documented by the study center via the web-based electronic data capture system (EDC system) Clincase (Quadratek Data Solutions Ltd.) will be stored on servers in two data centers in Berlin. The primary data center hosts the production systems, while the secondary data center is used for daily backup, storage, and recovery. Both data centers are ISO-certified for quality management and information technology security. After the database is closed, the data will be exported and transferred to the servers of Essen University Hospital. The data in Quadratek’s data centers will be deleted.

All electronic data at Essen University Hospital is stored on physical or virtual servers, including a backup/recovery concept. The computers used for the project are protected against external access by a firewall configured by the Essen University Hospital computer center. The data is only stored on servers at Essen University Hospital; there are no further copies on other computers. Remote access to the data is not possible. Only those involved in the project have access to the data. To conduct the study, IT has established a role/authorization concept at the server and application level. Predefined roles/authorizations regulate access to patient/subject data in the study and protect it against unauthorized access. Clincase user accounts, including the assignment of user rights, are created by a defined group of people at IMIBE/ZKSE who have been authorized to do so by project management. A patient identification list, which can be used to link health-related data to the patient identification number if necessary, is kept exclusively by the respective study center. Access to this list is only permitted to authorized study personnel from the respective study team and, if necessary, to auditors or inspectors appointed by the authorities for quality control purposes. Only anonymized results are used in the final report on the clinical study. Scientific publications on the study are based exclusively on aggregated anonymous data, i.e., appropriate technical and other measures are taken to modify the data in such a way that individual persons cannot be identified. The study data to be archived is stored in a lockable cabinet for 10 years in order to comply with regulatory requirements.

### Patient and public involvement

A patient representative from the patient advisory board Essen was involved in the development of the BREATH study. To disseminate results, they will be published and presented at expert conferences. In addition, patient support groups will receive all the necessary information created by BREATH.

### Trial governance and oversight

The BREATH trial is overseen by a Trial Steering Committee (TSC) consisting of thoracic oncologists from the West German Cancer Center (WTZ), physicians from the Department of Palliative Medicine, and oncology specialists from the Ruhrlandklinik. The TSC meets every 2 weeks via Zoom to review recruitment progress, protocol adherence, safety considerations, and operational challenges. Decisions on trial conduct, safety-related issues, and the need for protocol adaptations are made jointly within the TSC and documented in meeting minutes. The coordinating center for the trial is located at the Department of Palliative Medicine, University Hospital Essen, under the leadership of Prof. Dr. med. Tewes, who serves as overall principal investigator and is responsible for trial governance, ethical compliance, and oversight of daily study operations. The sport-scientific direction (Dr. Götte and Dr. De Lazzari) supervises intervention delivery, quality assurance, and training fidelity. Oncological expertise is provided by Priv.-Doz. Dr. Wiesweg (WTZ), ensuring clinical appropriateness and oncological safety oversight. Methodological and statistical oversight is provided by Prof. Dr. and Dr. Hüßler (IMIBE/ZKSE), who support data quality monitoring and statistical integrity. Additional clinical expertise from the cardiology department (Prof. Dr. Totzeck, PD. Dr. Mincu) informs safety considerations in patients with complex cardiovascular profiles. All listed investigators contribute to trial governance according to their domain.

### Data management

The BREATH study team will work closely with the Institute for Medical Informatics, Biometry and Epidemiology (IMIBE) and the Centre for Clinical Trials Essen (ZKSE). All study data will be documented on standardized case report forms and recorded with a good clinical practice (GCP)-compliant clinical data management system. The data will be checked for completeness and plausibility during the entire study period in order to guarantee a high data quality. The study database created for this purpose minimizes invalid data by performing automatic plausibility checks and highlighting missing values.

## Discussion

The BREATH trial addresses the unmet need for targeted exercise therapy interventions for advanced NSCLC patients, considering the challenges posed by the disease and its treatment. The proposed trial design and patient involvement strategies aim to address existing gaps in the literature and enhance the understanding of the potential cardiopulmonary benefits of exercise therapy in this specific patient population. Furthermore, our study design offers the benefits of having a control group while all patients will be receiving exercise therapy. Especially lung cancer patients are not aware of the potential benefits of exercise and maintaining physical activity during palliative treatment [84, 85]. In palliative treatment settings, longer or more intensive training sessions can be a barrier for patients. Therefore, it needs to be assessed whether a shorter exercise intervention focusing on endurance parameters can generate improved adherence or even have a more positive influence on patient-relevant endpoints, enhancing both quality of life and maximum oxygen uptake through combined endurance and strength training. BREATH can spread awareness based on the results in this specific population of advanced NSCLC patients. In addition, the high amount of comorbidities can be directly linked with shorter survival time and increased costs for therapies. Furthermore, the BREATH study offers insights of exercise therapy during active immunotherapy treatment with a sufficient sample size. Several theoretical and mouse model studies have highlighted the potential of combining exercise therapy with immunotherapy, although there is a lack of evidence from clinical trials [86, 87]. Currently, multiple trial protocols are listed on clinicaltrials.gov aiming to investigate the impact of exercise therapy during immunotherapy, including NCT06026111 [88], NCT04866810 [89], NCT06152926 [90], NCT04645680 [91] and NCT04263467 [92]. The ERICA study currently investigates the effects of aerobic exercise within 1 h prior to immunotherapy (pembrolizumab) in combination with chemotherapy (platinum-based) in metastatic NSCLC patients. The ERICA study will investigate the impact of aerobic exercise on immune biomarkers (NK cells, B lymphocytes, T lymphocytes, monocytes, subpopulations of dendritic cells on frozen PBMC, plasma biomarkers of sarcopenia and inflammation) [93].

To complement clinical and physiological outcomes, the BREATH trial also includes a structured evaluation of the intervention from the patient perspective. By combining quantitative satisfaction surveys and qualitative interviews, the evaluation provides important insights into feasibility, acceptance, and individual experiences with the intervention. These findings will help refine future exercise programs for this vulnerable population.

Consequently, BREATH has the potential to contribute evidence to the emerging field of immunotherapy combined with exercise therapy in a vulnerable patient cohort defined by high symptom burden and low quality of life.

## Trial status

Recruitment did start on 1 June 2024. The first patient was enrolled on June 12, 2024. As of the current date (August 27, 2025), a total of 25 patients have been enrolled in the BREATH study. Estimated end of enrollment/recruitment is planned for December 2026. Protocol version 1.1 date: 27.08.2025.

## Data Availability

Not available at this point.

## Abbreviations

COPD: Chronic obstructive pulmonary disease
NSCLC: Non-small-cell lung cancer
UICC: Union for International Cancer Control
ICI: Immune checkpoint inhibitors
PD-1: Programmed cell death protein 1
CTLA-4: Cytotoxic T-lymphocyte-associated protein 4
NMR: Nuclear magnetic resonance
BNP: Brain natriuretic peptide
MACE: Major adverse cardiac event
h1-RM: Hypothetical one-repetition maximum
CPET: Cardiopulmonary exercise testing
ITT: Intention-to-treat
PP: Per-protocol
DSS: Sodium trimethylsilylpropanesulfonate
PCA: Principal component analysis
PLS: Partial least squares
IMIBE: Institute for Medical Informatics, Biometry and Epidemiology
ZKSE: Centre for Clinical Trials Essen
GCP: Good clinical practice
